# Alterations of the serum and CSF ferritin levels and the diagnosis and prognosis of amyotrophic lateral sclerosis

**DOI:** 10.1016/j.ensci.2021.100379

**Published:** 2021-11-17

**Authors:** Parastou Paydarnia, Mahsa Mayeli, Mahan Shafie, Elmira Agah, Seyede Anis Hasani, Maryam Rashidi Jazani, Payam Sarraf

**Affiliations:** a School of Medicine, Tehran University of Medical Sciences, Tehran, Iran; b Iranian Center of Neurological Research, Neuroscience Institute, Tehran University of Medical Sciences, Tehran, Iran; c NeuroTRACT Association, Students' Scientific Research Center, Tehran University of Medical Sciences, Tehran, Iran; d Department of Neurology, Imam Khomeini Hospital Complex, Tehran University of Medical Sciences, Tehran, Iran

**Keywords:** ALS, Ferritin, Serum, CSF

## Abstract

**Background:**

The ALS diagnostic challenges necessitate more robust diagnostic and prognostic methods. A potential biomarker in this regard is the alterations of ferritin levels in the serum and CSF of patients compared to controls.

**Methods:**

The CSF and serum ferritin levels were measured in 50 ALS cases and 50 control patients with predefined exclusion criteria. The ELISA method was utilized for laboratory measurement and was statistically analyzed using the SPSS.

**Results:**

Heightened serum ferritin levels in cases were not statistically significant. However, CSF ferritin levels were significantly higher in ALS patients (*P* < 0.001). Serum ferritin levels were significantly negatively correlated with the disease duration (*P* = 0.015) and were significantly positively correlated with the disease progression rate (DPR) (*P* = 0.012).

**Conclusion:**

Heightened CSF ferritin levels can be used for the diagnosis of ALS. The correlation between the serum ferritin levels with the DPR and its correlation with the disease duration suggests potential prognostic utilities.

## Highlights

• Elevated serum ferritin level in patients with ALS is insignificant.
• CSF ferritin levels are significantly higher in ALS patients.
• Serum ferritin levels are negatively correlated with the disease duration.
• Serum ferritin levels are positively correlated with the disease progression rate.

## 1 Introduction

Amyotrophic lateral sclerosis (ALS) is a neurodegenerative disease characterized by upper and lower motor neuron involvement that could coincide [1]. With the latest prevalence rate of 5.2 per 100,000 cases in the US [2], the disease remains a challenge both diagnostically and prognostically. One of the latest estimates regarding the geographical distribution of ALS has indicated an incidence of 0.89 per 100,000 py in East Asia and 0.79 per 100,000 in South Asia [3].

To the best of our knowledge, the exact mechanism of degeneration of neurons in ALS is not fully defined. Previous studies suggested oxidative injury, excitotoxic stimulation, aggregation and/or dysfunction of critical proteins, and genetic factors as potential mechanisms of neurodegeneration. Former works have suggested iron homeostasis-related disorder in ALS that prompted many works to investigate the potential utilities of this mechanism. Dysregulation of iron metabolism among ALS patients was investigated in previous studies [[4], [5], [6]]. Moreover, some former studies showed alteration of iron and ferritin levels in serum and CSF in patients with ALS [5,[7], [8], [9]]. This mechanism is justifiable by the role of oxidative stress mechanisms in the pathogenesis of motor neuron damage in ALS. This was primarily brought up based on the observation of antioxidant enzyme superoxide dismutase 1 (SOD-1) mutations in familial ALS [10]. The oxidative stress caused by a disturbance in iron distribution and the caused labile iron pool is suggested as one underlying mechanism causing neurodegeneration [11].

Studies have evaluated serum ferritin levels as an indicator of iron metabolism and suggested an increase in ALS patients compared to controls [[12], [13], [14]]. However, whether the higher level of serum ferritin is specific to ALS is yet ambiguous [1]. On the contrary, some works investigating the iron and ferritin levels indicated decreased serum iron and non-significant alterations in the ferritin levels [15]. Although several studies have suggested that serum ferritin might be able to predict the prognoses of ALS, to date, there are a few studies that investigated the effect of ferritin levels in the cerebrospinal fluid (CSF) on the progression of ALS [4].

Thus, besides the controversies among the studies that assessed serum ferritin level, there is little knowledge concerning CSF ferritin in ALS. On the other hand, applying certain protocols were suggested to eliminate the biases, among which the low number of ALS cases in most studies is a major problem [16].

Advantaging the great number of patients at the multidisciplinary referral clinics of ALS at the *Imam* Khomeini Hospital Complex (Tehran, Iran) and applying a standard protocol, this study was conducted to reinvestigate the alterations of ferritin levels in the serum and CSF of ALS patients compared to the controls in response to the present controversies.

## 2 Methods

### 2.1 Design and participants

This study was reported based on the Strengthening the Reporting of Observational Studies in Epidemiology (STROBE) statement. In this case-control study, we enrolled 50 ALS patients and 50 controls from other neurological disease patients while considering the exclusion criteria referred to the neurologic clinic of *Imam* Khomeini Hospital Complex between April 2018 and March 2019. All the ALS patients were diagnosed with either definite, probable, or possible ALS according to the revised El Escorial criteria by experienced neurologists. The control group contained patients who were diagnosed with increased intracranial pressure, while those meeting the exclusion criteria were removed from the study. The exclusion criteria for both cases and controls contained patients with iron metabolic diseases, tumors, acute or chronic inflammation, inflammatory neurological diseases, liver diseases, renal diseases, thyroid diseases, hemorrhagic or neoplastic CNS diseases, any infectious diseases, and patients who received nutrition support.

### 2.2 Clinical variables and laboratory tests

At the time of the visit, ALS patients' clinical characteristics were obtained, including age, gender, site of onset, and disease duration (months). The onset site was divided into bulbar or limb onset. ALS Functional Rating Scale-Revised (ALSFRSr) and disease progression rate (DPR) were determined simultaneously by neurologists who were blinded regarding the cases and controls. DPR was calculated by the following formula: [(48-ALFRSr score)/disease duration in months].

We analyzed serum and CSF ferritin levels in both ALS cases and control groups. For this purpose, after obtaining written consent, peripheral blood samples were taken by venipuncture between 8 am and 12 pm. Simultaneously, as part of the diagnostic evaluation, a lumbar puncture was performed by neurologists, and CSF samples were obtained. Within 2 h of sampling, a 5 mL blood sample was taken from each patient and centrifuged at 1060*g* at four °C for 10 min. Also, a 2 mL CSF sample was centrifuged at 75*g* at four °C for 5 min. The supernatants were stored at −80 °C. Serum and CSF ferritin levels were measured via the enzyme-linked immunosorbent assay (ELISA). Measurements were received according to the manufacturer's instructions. Optical density (OD) was measured at a wavelength of 450 nm via a BioTek Synergy 2 microplate reader (BioTek, Highland Park, TX, USA). Based on the standard concentrations and corresponding OD values, standard curve linear regression was performed, and the corresponding sample concentrations were obtained by including the OD value of each sample in the regression equation.

### 2.3 Statistical analysis

Data analyses were done using IBM SPSS Statistics for Windows, version 25.0 (Armonk, NY: IBM Corp.). We described normally distributed continuous variables as mean with standard deviation and median [interquartile range boundaries (IQR)] for non-normally distributed variables. Continuous variables were analyzed using independent samples *t*-test and ANCOVA. Categorical variables were presented as numbers (percentage) and compared by the chi-square test. Differences in the CSF and serum ferritin levels among the ALS and control groups were determined by analysis of covariance (ANCOVA) adjusted for age and gender. The correlation between either serum and CSF ferritin levels and disease duration, ALSFRS-r, and DPR was measured using Spearman's and Pearson's correlation tests. Moreover, the association between serum and CSF ferritin levels and types of onset site were measured by the Mann-Whitney *U* test. Statistical significance was considered as *P*-value less than 0.05.

### 2.4 Ethical considerations

This study was approved by the Tehran University of Medical Sciences' research and ethics committee based on the Declaration of Helsinki 2013 with the ethical registration code of IR.TUMS.IKHC.REC.1396.3895. All of the participants gave written informed consent for using their medical records for research purposes.

## 3 Results

The demographic data and clinical characteristics of ALS and control patients are shown in Table 1. There was a significant statistical difference between the two groups regarding age (*P* < 0.001). To avoid the effects of potential confounders such as age and gender, ferritin levels among ALS and control groups were analyzed using analysis of covariance (ANCOVA) after adjusting for age and gender.

**Table 1:** Demographic, clinical characteristics, and ferritin levels in ALS and control patients.

Characteristic^a^	ALS patients	Control patients	P value
N (M/F)	50 (25/25)	50 (16/34)	0.067
Age	52.3 ± 11.9	36.6 ± 11.7	<0.001
Site of onset (B/L)	39 (78%)/11 (22%)	–	–
Duration of disease (months)	18 [7.0–24.0]	–	–
ALSFRS-r	19.8 ± 7.7	–	–
Disease progression rate (DPR)	1.7 [1.0–3.6]	–	–
Serum ferritin (mcg/L)	151.5 ± 92.7	127.2 ± 88.1	0.887^b^
CSF ferritin (mcg/L)	8.1 ± 5.2	5.5 ± 4.1	0.001^b^

N: number of patients, M: male, F: female, B: bulbar onset, L: limb onset, ALSFRSr: amyotrophic lateral sclerosis functional rating scale-revised, ALS: amyotrophic lateral sclerosis, CSF: cerebrospinal fluid.

a Data are presented as mean ± standard deviation, median [percentile 25-percentile 75], or number (%).

b *P* values were reported adjusting for age and sex.

The level of serum and CSF ferritin were analyzed in ALS patients and controls. Serum ferritin level was 151.5 ± 92.7 in the ALS group and 127.2 ± 88.1 in the control group. After controlling for the effect of age and sex, no significant difference was found between the two groups regarding serum ferritin levels (*P* = 0.887). However, there was a significant increase in the CSF ferritin level in ALS patients (8.1 ± 5.2) compared with control patients (5.5 ± 4.1, *P* = 0.001) (Table 1).

**Fig. 1:**
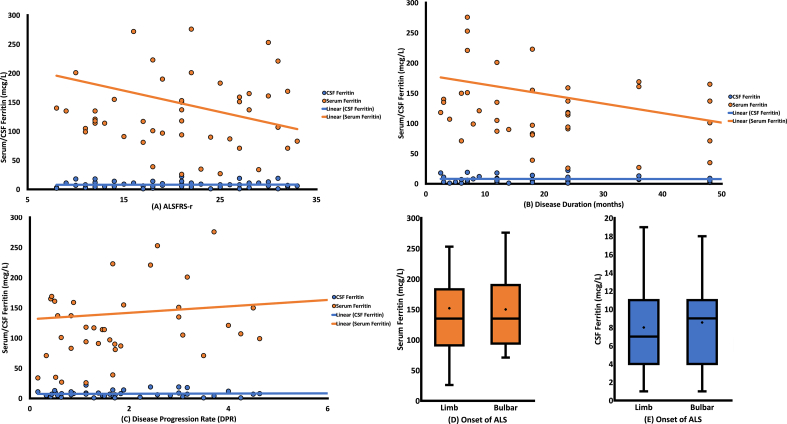
Correlation analyses of serum and CSF ferritin levels with ALSFRS-r, disease duration, and DPR (A–C). (A) shows that the levels of serum and CSF ferritin do not correlate with the ALSFRS-r score. (B) shows that serum ferritin level negatively correlates with disease duration (*p* = 0.015) and serum ferritin level negatively correlates with disease duration (*p* = 0.015), but CSF ferritin is not correlated with disease duration. (C) shows that serum ferritin level negatively correlates with DPR (*p* = 0.012), but CSF ferritin is not correlated with DPR. Serum and CSF ferritin levels based on the onset of ALS (D–E). The figure shows the level of ferritin in serum (D) and CSF (E) of limb and bulbar onset ALS patients. Serum and CSF ferritin levels had no significant differences regarding the onset of ALS.

The correlation between ferritin levels in serum and CSF of ALS patients and disease duration, ALSFRSr score, and DPR are presented in Table 2. Regarding the serum ferritin level, it was positively correlated with DPR (*P* = 0.012, *r* = 0.0361) and negatively with disease duration (*P* = 0.015, *r* = −0.348) but not correlated with ALSFRSr score (*P* = 0.268, *r* = −0.161). Furthermore, we did not find any significant association between CSF ferritin level and disease duration (*P* = 0.708, *r* = −0.055), ALSFRSr score (*P* = 0.969, *r* = 0.006), and DPR (*P* = 0.814, *r* = 0.035). Besides, we evaluated serum and CSF ferritin levels in either bulbar-onset or limb-onset ALS separately. There was no statistical difference between the site of onset (bulbar or limb onset) of ALS patients with ferritin levels in the serum and CSF (*P* = 0.888, *P* = 0.589, respectively) (Table 2). Graphical representations of these results are available in Fig. 1.

**Table 2:** Association between ALS clinical characteristics and serum and CSF ferritin levels.

	Serum ferritin		CSF ferritin	
	r_s_ / M [IQR]^a^	P value	r_s_ / M [IQR]^a^	P value
Site of onset		0.888		0.589
Bulbar onset	135 [94–190]		9 [4–11]	
Limb onset	135 [91–183]		7 [4–11]	
Duration of disease	−0.348	**0.015**	−0.055	0.708
ALSFRS-r score	−0.161	0.268	0.006	0.969
Disease progression rate (DPR)	0.361	**0.012**	0.035	0.814

ALSFRS-r: amyotrophic lateral sclerosis functional rating scale-revised.

Numerical variables were compared using spearman correlation test, while categorical variables were compared using the Mann-Whitney *U* test.

Significant *P*-values are bold.

a r_s_, the Spearman's rank correlation coefficient; M [IQR], median [interquartile range].

Finally, we determined the association between serum and CSF ferritin levels in the ALS group. There was no significant correlation between these two levels in ALS patients (*P* = 0.195, *r* = 0.188).

## 4 Discussion

This study was conducted to investigate the alterations of ferritin in the CSF and serum of ALS patients compared to controls and to assess their potential utilities as diagnostic and prognostic determination tools.

Our results indicated higher serum ferritin levels in cases compared to controls; however, this correlation yielded statistically insignificant after adjusting for covariates. Regarding the CSF ferritin levels, however, a statistically significant increase was observed in cases compared to those of controls. Since the current literature in this regard is still controversial, no deterministic results can be derived. One recent work with 435 sporadic ALS cases has suggested significantly increased serum ferritin levels in ALS cases compared to multiple system atrophy patients (MSA) and controls while reporting similar CSF ferritin levels between ALS patients with MSA cases and controls [14]. On the other hand, another recent work suggested significantly increased CSF ferritin levels in ALS patients compared to controls, while among the serum markers, the only significant result was regarding the heightened serum transferrin levels in ALS patients [4]. A very recent meta-analysis concluded significantly increased serum ferritin levels in ALS patients compared to controls [17], even though, according to this work, results regarding alterations of the CSF ferritin levels in the ALS patients remain a subject to debate [18].

Another remarkable finding in our study was a significant positive correlation between the increase in the serum ferritin level with the DPR and a significant negative correlation between the serum ferritin levels and the disease duration. Former works have confirmed this result; that is, increased serum ferritin level is associated with a higher progression rate [19] and a shorter disease duration [9]. Also, pooled hazard ratios of a noteworthy recent meta-analysis in this regard has indicated significantly reduced survival associated with heightened serum ferritin levels [17]. Altogether, these results indicate an association between elevated serum ferritin levels and a more aggressive manifestation of ALS, which might as well justify its negative correlation with the disease duration. These results are noteworthy due to the present ALS diagnostic challenges and might help in certain clinical conditions concerning diagnosis and prognosis determination [20]. However, more accurate designs and larger sample sizes are to be conducted longitudinally to present a more detailed overview of serum ferritin alterations in ALS and potentially etiological notions.

To the best of our knowledge, merely few works have so far addressed the associations between serum and CSF ferritin levels in regard to ALSFRSr, disease duration, and DPR, simultaneously with an acceptable sample size [1,4,9,[12], [13], [14],^16^] and our work adds to the evidence in favor of a significant association between heightened serum ferritin levels with disease duration and DPR, and also indicates that CSF ferritin levels are significantly elevated in patients with ALS. Also, our result regarding the increased serum ferritin levels in patients with ALS was insignificant, to the contrary of former works [14]. A future systematic review and Meta-analyses would be useful in evaluating the significance of this result.

## 5 Conclusion

In conclusion, our results indicate significantly higher CSF ferritin levels in ALS patients compared to controls that set the ground for further considerations of this as a diagnostic biomarker. Also, the significant positive correlation between the serum ferritin levels with the DPR and its significant negative correlation with the disease duration brings up the potential prognostic determination utility as a biomarker. Since the diagnostic difficulties of ALS remain challenging for many clinicians and considering the simplicity of measuring the alterations of ferritin levels compared to other potential biomarkers [21], further studies combining the current measurements with accuracy analysis are required to sum up the literature in this regard and to inquire the potential of determining diagnostic and prognostic cutoffs.
